# Influence of Government Price Regulation on the Price, Volume and Spending of Antibiotics in China: A Controlled Interrupted Time Series Study

**DOI:** 10.34172/ijhpm.2020.113

**Published:** 2020-07-18

**Authors:** Haishaerjiang Wushouer, Zhenhuan Luo, Xiaodong Guan, Luwen Shi

**Affiliations:** ^1^Center for Strategic Studies, Chinese Academy of Engineering, Beijing, China; ^2^School of Medicine, Tsinghua University, Beijing, China.; ^3^International Research Center for Medicinal Administration (IRCMA), Peking University, Beijing, China.; ^4^Department of Pharmacy Administration and Clinical Pharmacy, School of Pharmaceutical Sciences, Peking University, Beijing, China.

**Keywords:** Price Regulation, Interrupted Time Series, Antibiotics, China

## Abstract

**Background:** Chinese government established maximum retail prices for antibiotics listed in China’s National Reimbursement List in February 2013. This study aimed to analyze the impact of pharmaceutical price regulation on the price, volume and spending of antibiotics in China.

**Methods:** An interrupted time series design with comparison series was used to examine impacts of the policy changes on average daily cost, monthly hospital purchase volume and spending of the 11 price-regulated antibiotics and 40 priceunregulated antibiotics in 699 hospitals. One intervention point was applied to assess the impact of policy.

**Results:** After government price regulation, compared to price-unregulated antibiotics, the average daily cost of the price-regulated group declined rapidly (β=-5.68, *P*<.001). The average hospital monthly purchase spending of priceregulated antibiotics also decreased rapidly (β=-0.49, *P* <.010) and a positive trend change (β=0.04, *P*<.001) in average hospital spending of price-unregulated antibiotics was found.

**Conclusion:** Government regulation can reduce the prices and spending of price-regulated antibiotics. To control increasing expenditure, besides price caps regulation, factors determining drug utilization also need to be considered in policy designing.

## Background

**Key Messages**

** Implications for policy makers**

- Price caps can reduce prices of price-regulated medicines.
- To control increasing expenditure, factors determining drug utilization also need to be considered in policy designing.

** Implications for the public**

Evidences indicated that government price regulations exerted short-run effects on pharmaceutical prices, reducing them significantly right after the implement of the policy. Along with the prices, pharmaceutical expenditures also decreased rapidly in the short term. However, positive trend changes were observed in volume and spending of price-unregulated antibiotics after the regulation. This may indicate that the substitution between unregulated medicines and regulated medicines occurred after the price regulation.

Globally, the increase of pharmaceutical expenditure accounted for a significant share of overall healthcare spending.^1^ These issues posed serious challenges to policy-makers around the world.^2^ In response, varieties of regulatory controls, such as price ceiling and reference pricing, were implemented.^3-5^ Some studies suggested that regulations, such as price caps, played an essential role in curbing spending.^5-8^ However, some researchers reported that ineffective regulations might create a barrier to dynamic competition in the market, eventually resulting in the loss of consumers’ welfare or serving the interests of the industry being regulated.^9-11^ There was no consensus about whether pharmaceutical price regulations are necessary.

Pharmaceutical prices have long been regulated in China, except from 1992 to 1996, when the Chinese government conducted market-oriented reforms and let the market set drug prices.^12^ In China, financing sources for public hospitals included government fiscal budgets, medical service charges, and revenues from drug sales.^13^ Along with the decreasing government subsidies that forced hospitals to earn more revenue by increasing the volume of prescribing and drug sales, market-based pricing became a breeding ground for problems such as price increases, kickbacks, and corruption.^13^ To restrain continuous growth of pharmaceutical expenditure, the National Development and Reform Commission (NDRC) adopted price caps using a cost-plus calculation for each medicine listed in the National Reimbursement Drug List to reduce drug prices since 1998.^14^ Before all the pharmaceutical price ceiling policies were abolished in 2015, the NDRC used price caps to reduce medicine prices for 31 times that involved 1029 medicines by generic name.^15,16^

Another severe problem driven by increasing drug prices and financial incentives was irrational prescribing, especially over-prescribing of antibiotics.^17^ About 70% of prescriptions contained antibiotic drugs, of which the expenditure was the highest of any medicine in China.^18,19^ The NDRC set maximum retail prices for 29 antibiotics listed in the 2009 National Reimbursement Drug List in February 2013.^20^ The aim of this study was to analyze the impact of pharmaceutical price regulation on daily cost, volumes and spending of antibiotics in China.

## Methods

###  Study Design

An interrupted time series design with an intervention point was applied to assess the impact of price policy in this study. The intervention point, February 2013, served to assess the impact of government price cap regulation that was announced on December 31, 2012 and came into effect on February 1, 2013 on the average daily cost, average hospital purchase volume and average hospital purchase spending of the study medicines. More specifically, we included 11 antibiotics for systemic use out of 29 antibiotics regulated in February 2013 as price-regulated group and 40 antibiotics not listed in the National Reimbursement Drug List, and thus not subject to price caps during the study period as price-unregulated group (Supplementary file 1). Other policy changes through the study period remained substantially the same to these two groups of antibiotics. Though we used Anatomical Therapeutic and Chemical (ATC) classification J01 (ie, antibacterial for systemic use) as our inclusion criteria to include antibiotics in both groups, the differences of antibiotics between two groups would introduce new biases.

###  Data Source

Monthly data between January 2011 and March 2015 were extracted from the Chinese Medical Economic Information database of public hospital drug purchasing records.^21^ We searched all antibiotics in the database according to ATC classification J01,^22^ and extracted data for 51 antibiotics from 699 hospitals, including 476 tertiary hospitals, 217 secondary hospitals and 6 primary health facilities in 28 of the 31 provinces in China, which respectively accounted for 22.4%, 2.9% and 0.0007% of tertiary, secondary and primary public hospitals in 2015.^23^ Information extracted for each product comprised International Nonproprietary Names, dosage form, strength, manufacturer, monthly drug purchase price per package, monthly purchase volumes and monthly hospital spending.

###  Outcome Measures

The primary outcome was the average daily cost, calculated based on defined daily dose (DDD).^24^ Secondary outcomes of interest were average hospital purchasing volume (numbers of DDD) and average spending of the 11 price-regulated drugs and 40 price-unregulated drugs. All price and spending data were adjusted to January 2011 using the consumer price index for healthcare (National Bureau of Statistics of China, 2019) and reported in US dollar based on the 2011 exchange rate (CNY = Chinese Yuan, 1 CNY = 0.155 US$ in 2011).^25^

 Aside from assessing outcomes over time for both groups, we also modeled the intervention effects using monthly differences in the outcomes in two groups to estimate the relative impact of regulation on the regulated products, controlling for any other externalities that may affect the outcomes in the price-unregulated group products.

###  Statistical Analysis

 Interrupted time series analysis and segmented linear regression models were used to estimate levels and trends of the outcomes in the pre-intervention periods, as well as changes in levels and trends in the post-intervention periods. Segmented linear regression models with two interruption points were formulated to detect the effect on daily cost, hospital monthly purchase volume and spending, as in equation (1):


(1) $$Y_{it}=\beta_{0}+\beta_{1}\times time_{t}+\beta_{2}\times regulation+\beta_{3}\times reg\_trend+\varepsilon_{it}$$

*β*_0 _denoted the baseline; *β*_1 _denoted the pre-regulation trend; *β*_2 _denoted the change in level after the regulation policy; *β*_3 _denoted the change in trend after the regulation policy; Key coefficients were *β*_2 _and *β*_3._ To estimate the combined level and trend effects of the policy changes, we calculated the absolute difference in *Y*_it_ at 12 months after regulation, compared to the counterfactual, that is, the estimated *Y*_it_ had the intervention not happened.

 The Durbin-Watson test was performed to estimate level of residual autocorrelations and the Cochrane-Orcutt auto-regression procedure was used to correct for first order serially correlated errors when needed. In addition, the Kolmogorov–Smirnov statistic was used for testing the normality of the residuals. All analyses were performed using Stata 14.0.

## Results

###  Influence of Government Regulation on Average Daily Cost

**Figure 1:**
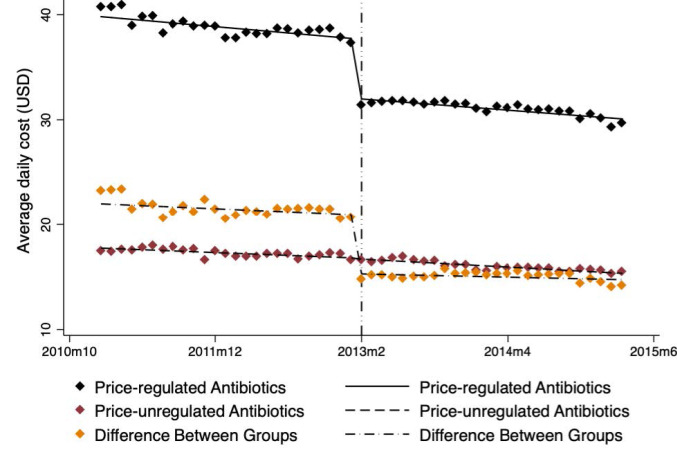
Influence of Government Price Regulation on Average Daily Cost Among Price-Regulated (n = 11), not Price-Regulated (n = 40) and Average Daily Cost Differences, 2011-2015.

The average daily cost declined over time in both price-regulated and price-unregulated antibiotic groups, from January 2011 to March 2015 (Table, Figure 1).

**Table:** Results of Interrupted Time Series Analyses of the Impact of Government Price Regulation on Daily Cost, Volume and Spending for Price-Regulated and Price-Unregulated Antibiotics, 2011-2015

	**Baseline Level **	**Baseline Trend**	**Post-regulation Level Change**	**Post-regulation Trend Change**	**Change at 12 Months After Regulation**
Average daily cost (USD)					
Price-regulated group	39.90***	-0.09***	-5.68***	NS	-5.56***
Price-unregulated group	17.78***	-0.04***	NS	NS	NS
Difference	22.05**	NS	-5.67***	NS	-5.41***
Average hospital purchasing volume (Thousand DDD)					
Price-regulated group	274.84***	4.65***	NS	NS	NS
Price-unregulated group	172.22***	-1.78**	NS	2.94**	45.22*
Difference	99.80***	6.65***	-37.13*	-3.09**	-74.21***
Average hospital purchase spending (million USD)					
Price-regulated group	2.11***	0.04***	-0.49**	NS	-0.73**
Price-unregulated group	1.81***	-0.03***	NS	0.04***	0.58**
Difference	0.28*	0.07***	-0.61***	-0.06***	-1.35***

Abbreviations: DDD, defined daily dose; NS, not significant. * *P* < .050; ** *P* < .010; *** *P* < .001; Price-regulated group: 11 antibiotics with price regulation in 2013. Price-unregulated group: 40 antibiotics without price regulation.

After government price regulation in February 2013, the average daily cost of the price-regulated group declined immediately (level change β = -5.68, *P* < .001) while the average daily cost of price-unregulated group showed no significant change. No significant trend change was observed in both groups. At 12 months after the regulation, there was an estimated reduction in the average daily cost for price-regulated drugs of -5.56 (*P* < .001), with significant decline relative to price-unregulated drugs (level change β = -5.41, *P* < .001).

###  Influence of Government Regulation on Average Purchase Volumes 

**Figure 2:**
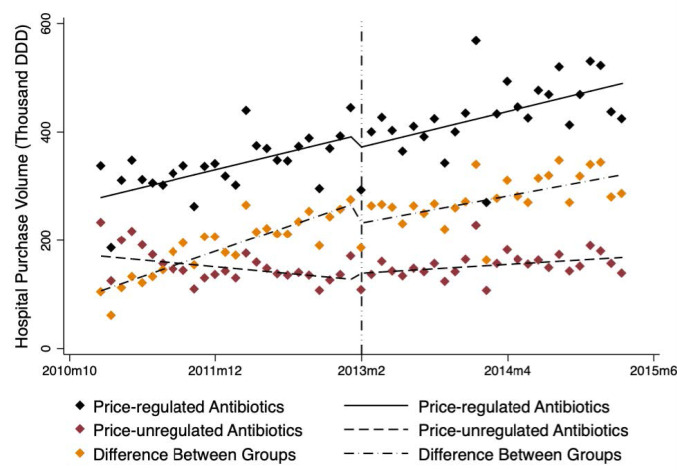
Influence of Government Price Regulation on monthly purchase volumes Price-Regulated (n = 11), not Price-Regulated (n = 40) and monthly group Differences, 2011-2015.

From January 2011 to March 2015, the average purchase volumes of price-regulated drugs demonstrated an increasing trend while the average purchase volumes of price-unregulated drugs basically remained stable relative to the price-unregulated group (Figure 2).

The only significant change in level and trend of average purchase volumes was the trend changes in price-unregulated drugs after government price regulation in February 2013 (trend change β = 2.94, *P* < .010). Except that, there was no statistically significant change in neither group after regulation. At 12 months after the regulation, there was an estimated increase in average purchase volumes for price-unregulated drugs (β = 45.22, *P* < .050), narrowing the gap between two groups (-74.21 thousand DDD, *P* < .001).

###  Influence of Government Regulation on Average Hospital Spending

**Figure 3:**
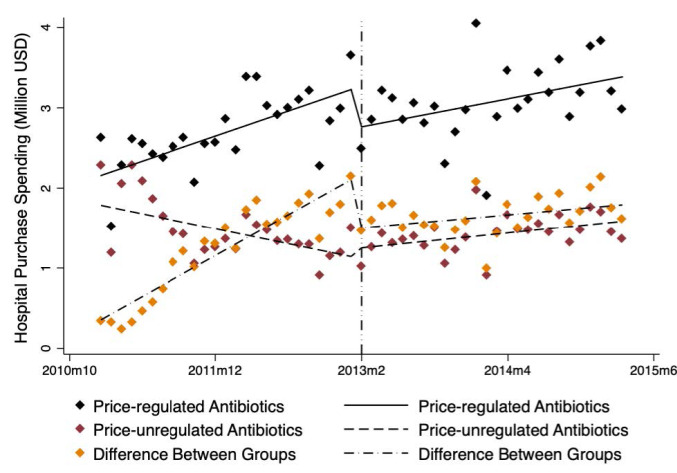
Influence of Government Price Regulation on monthly spending on price-regulated (n = 11), not Price-Regulated (n = 40) and monthly group Differences, 2011-2015.

The average hospital spending of price-regulated drugs showed an increasing trend while the average hospital spending of price-unregulated drugs basically remained stable relative to the price-regulated group from January 2011 to March 2015 (Figure 3).

Unlike the average purchase volumes of price-regulated group that had no significant change after regulation, the average hospital spending of price-regulated drugs decreased rapidly (level change β = -0.49, *P* < .010). Moreover, a positive trend change (trend change β = 0.04, *P* < .001) was observed in average hospital spending of price-unregulated drugs after regulation. At 12 months after regulation, the absolute spending difference between the groups was significantly lower (US$-1.35 million, *P* < .001) than would have been expected without the regulation.

## Discussion

 This study showed that the introduction of government maximum retail price regulation was associated with rapid decrease in average daily cost and spending of price-regulated antibiotics. Additionally, we observed positive trend changes in average hospital volume and spending of price-unregulated antibiotics after the regulation.

The rapid decrease in average daily cost and spending of price-regulated antibiotics confirmed conclusions drawn from aforementioned studies in China and also in other therapeutic areas.^26-28^ Evidences indicated that government price regulations exerted short-run effects on pharmaceutical prices, reducing them significantly right after the implement of the policy. Along with the prices, pharmaceutical expenditures of price-regulated antibiotics also decreased rapidly in the short term.

However, we observed positive trend changes in average hospital volume and spending of price-unregulated antibiotics after the regulation. The behaviors that physicians or hospitals substitute unregulated medicines for regulated medicines to maintain their income level might contribute to this phenomenon.^26,29^ In China, to support health providers’ normal operations, the drug mark-up policy was put forward which allows hospitals to set a fixed percent mark-up up to 15% on the wholesale prices of drugs.^30^ Thus, the increasing volume of unregulated drug might be in response to the kickback’s compression of regulated drugs after government price regulation. In this study, the evidences of cefpirome and cefoselis were found to support the substitution speculation. Along with the decreasing volume of cefpirome (one of price-regulated drugs), the volume of cefoselis (one of price-unregulated drugs) was continuously increasing during our study period (both two drugs are the fourth generation cephalosporins and their daily cost were similar before the regulation). Additionally, the results could be correlated to the composition of price-unregulated group. Among 40 antibiotics in the price-unregulated group, 19 were cephalosporins. Studies showed that cephalosporins became the most consumed antibiotic class of total antibiotic consumption in China.^31,32^ Other than different disease spectrums, one possible explanation might be that cephalosporin was recommend by national guidance for majority of the perioperative prophylaxis in China.^33^

Furthermore, this study also indicated a nonnegligible challenge to maximum retail price regulation policies. Although price caps can efficiently curve regulated antibiotic price in the short term, positive trends in hospital volume and spending would eventually let the bullet of maximum retail price regulation policy miss its target, that is, to constrain medicine expenditure. Apart from price regulations, factors determining drug utilization also need to be considered in the process of policy designing. Since the 2009 health system reform, the Chinese government has been committed to tackling the irrational use of antibiotics by enhancing antimicrobial stewardship.^34^ The Ministry of Health implemented a decree including comprehensive regulations on antibiotics, updated the national guidelines for antibiotic use in clinical practice, and mandated hospitals to regularly review and evaluate antibiotic prescription.^35-38^ Additionally, the zero mark-up policy theoretically removed the 15% profit margin from drug sales, eliminating the financial incentive of over-prescribing.^39^ All these efforts jointly built up a regulatory and fiscal framework to curb growing pharmaceutical expenditure and irrational use of antibiotics.

###  Limitation

 This study had several limitations. First, the full list of products under government price regulation since 1996 was unable to obtain. The 11 price-regulated antibiotics selected as price-regulated group in this study may not be representative of all products, which could lead to selection bias. Efforts are also needed towards studies in different policy settings with more representative samples. Second, the baseline data of two groups in this study was different in many ways. However, the baseline trends of average daily cost were quite similar, suggesting that differential changes observed following the government pricing policies were indicative of true differences. Third, given that our analyses are based on aggregated procurement data, we have no information on indications of use and potential therapeutic substitution and cannot assess impacts of individual product generic and brand status.

## Conclusion

 We found that the maximum retail price policy in China had an immediate reduction effect on the prices and the spending of price-regulated antibiotics, while it failed to change the upward trend of the price-regulated antibiotics spending. Isolated price control policy is not effective enough to constrain the rapid growth of medicine expenditure because the pharmaceutical expenditures are not only determined by drug prices. Other cooperative policies focused on rational drug utilization are needed.

## Acknowledgements

 All authors are grateful to staff of Chinese Pharmaceutical Association for their support and cooperation in data access and analysis. The contents are solely the responsibility of the authors, and do not reflect the views of the funding bodies or any organization.

## Ethical issues

 We used secondary data from Chinese Medical Economic Information database. As such, ethical approval was not required.

## Competing interests

 Authors declare that they have no competing interests.

## Authors’ contributions

 LS and XG conceptualised and designed the study. ZL contributed to analysis of the data. XG, HW, and ZL conducted the final analyses. HW and ZL drafted the initial manuscript. All authors contributed to the critical revision of the manuscript and approved the final version.

## Funding

 This work was supported by National Natural Science Foundation of China (grant No.71774005). The funders had no role in study design, data collection and analysis, decision to publish, or preparation of the manuscript.

## Availability of data and materials

 The data that support the findings of this study are available from Chinese Medical Economic Information database but restrictions apply to the availability of these data, which were used under license for the current study, and so are not publicly available. Data are however available from the authors upon reasonable request and with permission of Chinese Pharmaceutical Association.

## Authors’ affiliations

^1^Center for Strategic Studies, Chinese Academy of Engineering, Beijing, China ^2^School of Medicine, Tsinghua University, Beijing, China. ^3^International Research Center for Medicinal Administration (IRCMA), Peking University, Beijing, China. ^4^Department of Pharmacy Administration and Clinical Pharmacy, School of Pharmaceutical Sciences, Peking University, Beijing, China.

## Supplementary files


Supplementary file 1 contains Tables S1-S3.

## References

[1] Belloni A, Morgan D, Paris V. Pharmaceutical Expenditure and Policies. Paris: OECD Publishing; 2016. 10.1787/5jm0q1f4cdq7-en

[2] OECD. Pharmaceutical Expenditure. OECD; 2010:132-133.

[3] Ess SM, Schneeweiss S, Szucs TD (2003). European healthcare policies for controlling drug expenditure. Pharmacoeconomics.

[4] Borrell JR (2011). Drug price regulation: recent trends and downstream neglected issues.

[5] Sood N, de Vries H, Gutierrez I, Lakdawalla DN, Goldman DP (2009). The effect of regulation on pharmaceutical revenues: experience in nineteen countries. Health Aff (Millwood).

[6] Rane W (1998). Price Control on Drugs Is Essential. Economic and Political Weekly.

[7] López-Casasnovas G, Puig-Junoy J (2000). Review of the literature on reference pricing. Health Policy.

[8] Brekke KR, Grasdal AL, Holmås TH (2009). Regulation and pricing of pharmaceuticals: reference pricing or price cap regulation?. Eur Econ Rev.

[9] Stigler GJ (1971). The theory of economic regulation. Bell J Econ Manage Sci.

[10] Miziara NM, Coutinho DR (2015). Problems in the regulatory policy of the drug market. Rev Saude Publica.

[11] Vaidyanathan G. Poor May Suffer as India Caps Drug Prices. Nature; 2012. https://www.nature.com/news/poor-may-suffer-as-india-caps-drug-prices-1.12046. Accessed June 5, 2019.

[12] Sun Q, Santoro MA, Meng Q, Liu C, Eggleston K (2008). Pharmaceutical policy in China. Health Aff (Millwood).

[13] World Bank. Fixing the Public Hospital System in China. Washington, DC: World Bank; 2010.

[14] National Development and Reform Commission. Notice on the Government Pricing Scheme for Medicines. http://zwgk.gd.gov.cn/006939828/201308/t20130830_399800.html. Accessed June 5, 2019. Published 2003.

[15] Luk S (2015). The politics of drug price control policy in China: regulation, deregulation and Re-regulation. J Contemp East Asia Stud.

[16] National Development and Reform Commission. List of Priced Drugs of the National Development and Reform Commission. http://www.ndrc.gov.cn/fzgggz/jggl/zcfg/200508/t20050802_747962.html. Accessed March 25, 2019.

[17] Li Y, Xu J, Wang F (2012). Overprescribing in China, driven by financial incentives, results in very high use of antibiotics, injections, and corticosteroids. Health Aff (Millwood).

[18] Guan X, Liang H, Xue Y, Shi L (2011). An analysis of China’s national essential medicines policy. J Public Health Policy.

[19] Han S, Liang H, Su W, Xue Y, Shi L (2013). Can price controls reduce pharmaceutical expenses? a case study of antibacterial expenditures in 12 Chinese hospitals from 1996 to 2005. Int J Health Serv.

[20] National Development and Reform Commission. Notice on the Government Pricing Scheme for Immune system, Anti-cancer and Blood system Medicines. http://www.ndrc.gov.cn/fzgggz/jggl/zcfg/201209/t20120918_505462.html. Accessed September 6, 2019.

[21] Science and Technology Development Center of Chinese Pharmaceutical Association. Brief Introduction to CMEI. http://www.cmei.org.cn/list/?343_1.html. Accessed February 14, 2020.

[22] WHO Collaborating Centre for Drug Statistics Methodology. Guidelines for ATC Classification and DDD Assignment. https://www.whocc.no/filearchive/publications/2019_guidelines_web.pdf. Accessed July 23, 2019. Published 2019.

[23] Yearbook of Chinese Health Statistics in 2015. China Ministry of Health; 2016.

[24] Defined daily dose (1995). Pharmacoecon. Outcomes News.

[25] National Bureau of Statistics of China. Time Series Data -- Annual Data: The Exchange Rate Between RMB and USD. http://data.stats.gov.cn/english/easyquery.htm?cn=C01. Accessed November 20, 2019.

[26] Meng Q, Cheng G, Silver L, Sun X, Rehnberg C, Tomson G (2005). The impact of China’s retail drug price control policy on hospital expenditures: a case study in two Shandong hospitals. Health Policy Plan.

[27] Dong Z, Liu G, Wu J, Wu J (2008). Effects of drug price control policies on antibiotics: evidence from Beijing. China Journal of Pharmaceutical Economics.

[28] Guan X, Wushouer H, Yang M (2019). Influence of government price regulation and deregulation on the price of antineoplastic medications in China: a controlled interrupted time series study. BMJ Open.

[29] Yip WC, Hsiao W, Meng Q, Chen W, Sun X (2010). Realignment of incentives for health-care providers in China. Lancet.

[30] China National Health Accounts Report. China National Health Development Research Center; 2009.

[31] Wushouer H, Tian Y, Guan XD, Han S, Shi LW (2017). Trends and patterns of antibiotic consumption in China’s tertiary hospitals: Based on a 5 year surveillance with sales records, 2011-2015. PLoS One.

[32] Wushouer H, Zhou Y, Zhang X (2020). Secular trend analysis of antibiotic utilisation in China’s hospitals 2011-2018, a retrospective analysis of procurement data. Antimicrob Resist Infect Control.

[33] Department of Medical Administration and Committee of Experts on Rational Drug Use, National Health and Family Planning Commission of the People’s Republic of China. National Antimicrobial Therapy Guidance. 1st ed. Beijing: People’s Medical Publishing House Co Ltd; 2012.

[34] He P, Sun Q, Shi L, Meng Q (2019). Rational use of antibiotics in the context of China’s health system reform. BMJ.

[35] Xiao Y, Li L (2013). Legislation of clinical antibiotic use in China. Lancet Infect Dis.

[36] Yin J, Li Q, Sun Q (2018). Antibiotic consumption in Shandong Province, China: an analysis of provincial pharmaceutical centralized bidding procurement data at public healthcare institutions, 2012-16. J Antimicrob Chemother.

[37] China Minister of Health (2012). Regulations for clinical application of antibacterial agents (Decree No 23 Issued by the Minister of Health). Chinese Journal of Clinical Infectious Diseases.

[38] Xiao Y (2018). Antimicrobial stewardship in China: systems, actions and future strategies. Clin Infect Dis.

[39] Yin J, Wu C, Wei X, Sun Q (2018). Antibiotic expenditure by public healthcare institutions in Shandong province in China, 2012-2016. Front Pharmacol.

