# Supplementary material for: Influence of Government Price Regulation on the Price, Volume and Spending of Antibiotics in China: A Controlled Interrupted Time Series Study
Source: Int J Health Policy Manag. 2020 Jul 18;11(2):218–23. doi: 10.34172/ijhpm.2020.113 (PMC9278607; doi:10.34172/ijhpm.2020.113)
Supplement: Supplementary file 1 — contains Tables S1-S3. [file ijhpm-11-218-s001.pdf]

Supplementary file 1

Table S1. Antibiotics samples of price-regulated group

No.	Generic Name	ATC	Routine	Single-Supplier
1	biapenem	J01DH05	oral	generic only
2	cefpirome	J01DE02	injectious	generic only
3	cephathiamidine	J01DB	injectious	generic only
4	clarithromycin	J01FA09	oral	brand-name and generic
5	dirithromycin	J01FA13	oral	generic only
6	faropenem	J01DI03	injectious	generic only
7	levornidazole	J01XD03	injectious	generic only
8	linezolid	J01XX08	injectious	brand-name only
9	mezlocillin and sulbactam	J01CR	injectious	generic only
10	sulbactam	J01CG01	injectious	generic only
11	sulbenicillin	J01CA16	injectious	generic only

Table S2. Antibiotics samples of price-unregulated group

No.	Generic Name	ATC	Routine	Single-Supplier
1	amoxicillin and flucloxacillin	J01CR50	injectious	generic only
2	amoxicilin sulbactam pivoxil	J01CR02	oral	generic only
3	amoxicillin and dicloxacillin	J01CR	oral	brand-name and generic
4	antofloxacin	J01MA	injectious	brand-name only
5	ampicillin and probenecid	J01CR	oral	generic only
6	ampicillin and clixacillin	J01CR	oral	brand-name only
7	ertapenem	J01DH03	injectious	brand-name only
8	flucloxacillin	J01CF05	injectious	brand-name and generic
9	compound cefaclor	J01DC	oral	generic only
10	erythromycin cyclocarbonate	J01FA01	oral	generic only
11	gatifloxacin	J01MA16	injectious	generic only
12	thiamphenicol	J01BA02	oral	generic only
13	thiamphenicol glycinate	J01BA02	injectious	generic only

14	roxithromycin and ambroxol	J01FA06	injectious	generic only
15	midecamycin	J01FA03	injectious	generic only
16	pazufloxacin	J01MA18	injectious	generic only
17	sultamicillin	J01CR04	oral	generic only
18	sparfloxacin	J01MA09	oral	generic only
19	cefpodoxime proxetil	J01DC	oral	generic only
20	cefodizime	J01DD09	injectious	brand-name and generic
21	cefuroxime axetil	J01DC02	oral	brand-name and generic
22	cefmenoxime	J01DD05	injectious	generic only
23	cefamandole nafate	J01DC03	injectious	generic only
24	cefonicid	J01DC06	injectious	generic only
25	cefoperazone	J01DD12	injectious	brand-name and generic
26	cefoperazone and tazobactam	J01DD12	injectious	generic only
27	cefpiramide	J01DD11	injectious	brand-name and generic
28	cefadroxil and trimethoprim	J01DD	oral	generic only
29	ceftriaxone and tazobactam	J01DD	injectious	generic only
30	cephalothin	J01DB03	injectious	generic only
31	cefoselis	J01DE	injectious	generic only
32	cefotaxime and sulbactam	J01DD	injectious	generic only
33	ceftazidime and tazobactam	J01DD	injectious	generic only
34	cefetame pivoxil	J01DC	oral	generic only
35	cefteram pivoxil	J01DC	oral	brand-name and generic
36	ceftezole	J01DB12	injectious	brand-name and generic
37	cefditoren pivoxil	J01DC	oral	brand-name and generic
38	enoxacin	J01MA04	injectious	generic only
39	acetylkitasamycin	J01FA	oral	generic only
40	acetylmidecamycin	J01FA	oral	brand-name and generic

Table S3. 29 price-regulated antibiotics

Category	No.	Generic Name	ATC
A	1	sulbenicillin	J01CA16
	2	mezlocillin and sulbactam	J01CR
	3	sulbactam	J01CG01
	4	cephathiamidine	J01DB
	5	cefpirome	J01DE02
	6	biapenem	J01DH05
	7	faropenem	J01DG
	8	dirithromycin	J01FA
	9	clarithromycin	J01FA
	10	levornidazole	J01XD03
	11	linezolid	J01XX08
B	12	latamoxef	J01DD06
	13	ofloxacin	J01MA01
	14	cefmetazole	J01DC09
	15	cefminox	J01DC12
C	16	sulfadiazine zinc	D06BA
	17	compound sulfadiazine zinc	D06BA
	18	compound polymyxin b	S03AA30
	19	fluorometholone and gentamicin	S01AA
	20	natamycin	D01AA02
	21	tobramycin and dexamethasone	S01CA01
	22	capreomycin	J04AB30
	23	rifabutin	J04AB04
	24	rifamycin	S01AA16
	25	gemifioxacin	J01MA15
	26	pasiniazide	J04AC
	27	ethambutol hydrochloride-pyrazinamide-rifampicin-Isoniazid II	J04AM06
	28	ethambutol hydrochloride,rifampicin and isoniazid	J04AM07
	29	houttuynin	*

*Houttuynin [CH3(CH2)8COCH2CHO] is one of the main ingredients in the volatile oil of the Chinese herb *Houttuynia cordata* Thunb with no ATC code. Sodium houttuyfonate has been used in China for the clinical treatment of bronchitis and upper respiratory infection for many years.
